# When scientific paradigms lead to tunnel vision: lessons from the study of fear

**DOI:** 10.1038/s41539-017-0007-4

**Published:** 2017-03-27

**Authors:** Denis Paré, Gregory J. Quirk

**Affiliations:** 1 Center for Molecular and Behavioral Neuroscience, Rutgers State University, Newark, NJ 07102 USA; 2 University of Puerto Rico School of Medicine, San Juan, PR 00936-5067 USA

## Abstract

For the past 30 years, research on the amygdala has largely focused on the genesis of defensive behaviors as its main function. This focus originated from early lesion studies and was supported by extensive anatomical, physiological, and pharmacological data. Here we argue that while much data is consistent with the fear model of amygdala function, it has never been directly tested, in part due to overreliance on the fear conditioning task. In support of the fear model, amygdala neurons appear to signal threats and/or stimuli predictive of threats. However, recent studies in a natural threat setting show that amygdala activity does not correlate with threats, but simply with the movement of the rat, independent of valence. This was true for both natural threats as well as conditioned stimuli; indeed there was no evidence of threat signaling in amygdala neurons. Similar findings are emerging for prefrontal neurons that modulate the amygdala. These recent developments lead us to propose a new conceptualization of amygdala function whereby the amygdala inhibits behavioral engagement. Moreover, we propose that the goal of understanding the amygdala will be best served by shifting away from fear conditioning toward naturalistic approach and avoidance paradigms that involve decision-making and a larger repertoire of spontaneous and learned behaviors, all the while keeping an open mind.

Shortly before the first report implicating the amygdala in conditioned fear,^1^ the *Handbook of Physiology* published a comprehensive review on the amygdala. Authored by Pierre Gloor,^2^ this chapter critically summarized a now largely forgotten body of stimulation, recording, and lesion studies, including the well-known Kluver–Bucy syndrome.^3^ With respect to the latter, Gloor commented that while changes in emotional behaviors are consistently observed after amygdala lesions, most aspects of the Kluver–Bucy syndrome (other than the visual agnosia) could also be observed with lesions largely restricted to the amygdala. These included the indiscriminate urge to attend and respond to all environmental stimuli, to ingest non-food objects, or to seek sexual gratification undiscerningly. He wrote: “Obviously, all these behavioral mechanisms are intimately interlocked and the full syndrome is more than the sum of individual deficits attributable to individual anatomical structures” (see ref. 2, p. 1411).

Nevertheless, reinforced by a series of studies implicating the amygdala in the genesis of innate and learned fear,^1, 4, 5^ the view emerged that the emotional disturbances seen in the Kluver–Bucy syndrome—the apparent loss of fear—resulted from amygdala lesions, while the other anomalies depended on damage to neighboring structures. This fateful simplification led amygdala researchers to focus on fear, particularly learned fear, and Pavlovian fear conditioning emerged as the dominant experimental paradigm to study the amygdala.^6–9^

## Pavlovian fear learning: perspectives and untested assumptions

Early findings suggested that fear memory traces involve a simple circuit, residing entirely in the amygdala: information about the conditioned (CS) and unconditioned (US) stimuli converged onto lateral amygdala (LA) neurons, leading to a potentiation of CS inputs and the genesis of defensive behaviors via LA projections to the central amygdala (CeA). This arrangement inspired confidence that fear learning would be readily amenable to experimental scrutiny, potentially leading to insights in the basic mechanisms of memory and the causes of human anxiety disorders. These factors, combined with major investments from the National Institutes of Health, led to an explosion of publications that continues to this day. As a result, major progress has been made in characterizing the network, cellular, and molecular mechanisms supporting the early formation and subsequent consolidation of memory traces in the amygdala.

However, it has also become clear that fear learning involves a more intricate circuit than initially envisioned. For instance, it was found that the amygdala is not the only site of plasticity for Pavlovian fear: other areas also display increases in CS responsiveness that are critical for fear learning. These include amygdala-projecting auditory thalamic and cortical neurons^10, 11^ and, as recently shown, midline thalamic neurons.^12^ Even within the amygdala, it was found that multiple parallel inhibitory and excitatory circuits are differentially involved^13, 14^ and regulated by medial prefrontal neurons during the expression or extinction of conditioned fear.^15^ In addition, the same neurons and circuits that were thought to mediate learned fear have been shown to respond to rewards^16^ and support the acquisition of responses driven by positively valenced US.^17, 18^ Nevertheless, the study of fear learning sustained the early notion that activity of neurons in the amygdala serves to signal threat and in turn generates defensive behaviors. Surprisingly, this foundational assumption was not fully tested until recently.

## Peering outside the box

**Fig. 1:**
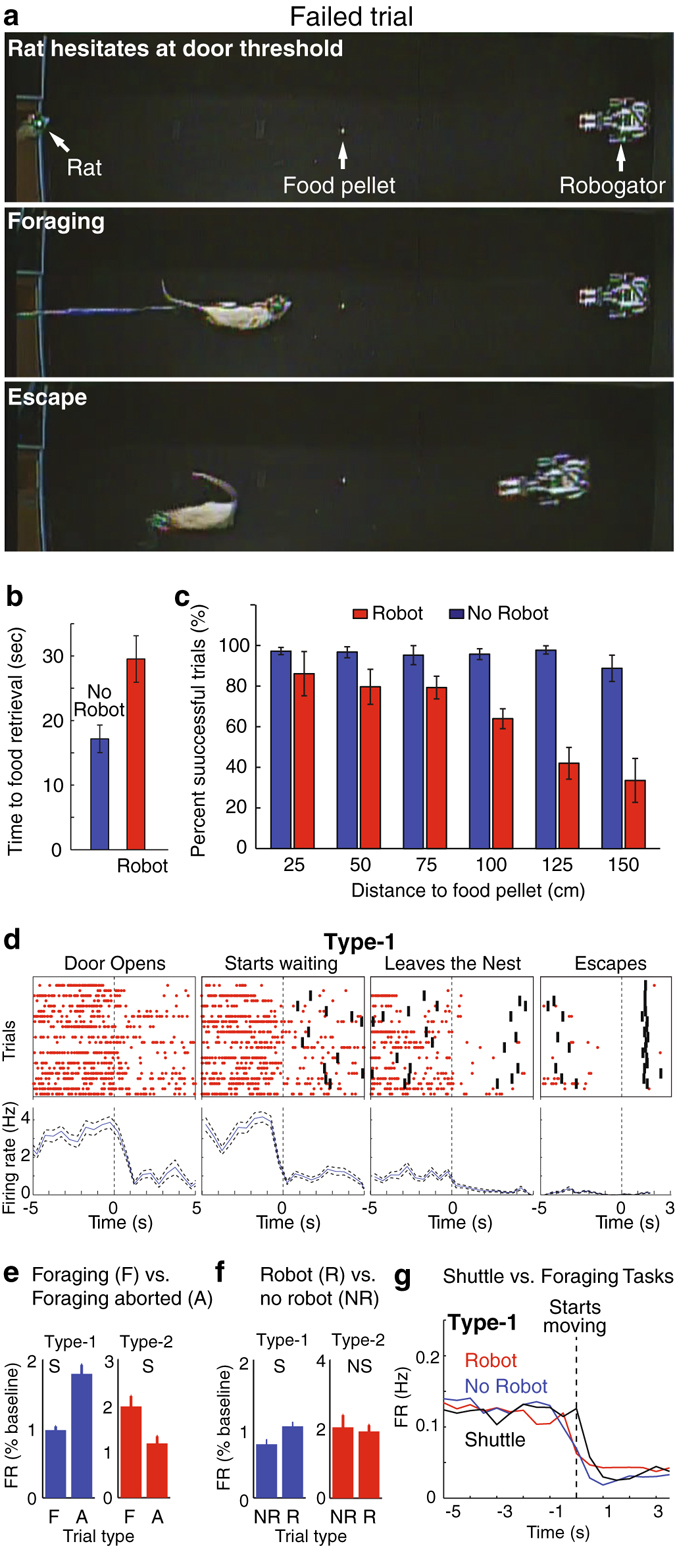
Foraging task. The foraging apparatus had two compartments divided by a door: a dimly lit nest and a much brighter foraging arena. After training rats to retrieve sweetened food pellets in the absence of Robogator, alternating trial blocks were conducted with or without the Robogator. Each trial started by opening the door. **a** Example of failed trial. **b** Time from door opening to food retrieval increases when the robot is present. **c** Proportion of successful trials is lower when the robot is present. *Red*, robot present; *blue* robot absent. **d** Activity of representative Type-1 cell during the foraging task. Only successful trials are shown. *Top* rasters (*dots* indicate spikes and *each line* is a trial). *Bottom* average of all available trials ± SEM. **e** Normalized FR of Type-1 (*blue*) and Type-2 (*red*) cells during waiting periods preceding initiation of foraging (F) or retreat into the nest (A). **f** Normalized FR of Type-1 and Type-2 cells during waiting periods in the presence (R) vs. absence (NR) of the Robogator. **g** FR of Type-1 cell in three conditions: during shuttle task (*black*), or during foraging task with Robot present (*red*) or absent (*blue*). *Vertical dashed line* indicates initiation of foraging. *S* significant, *NS* non-significant. *Source*: Ref. 24

Prior to the widespread adoption of freezing as an index of Pavlovian fear, other measures of defensive behaviors were used^19^ and fear was often studied in semi-naturalistic foraging settings.^20, 21^ After a long period of neglect, these ethological approaches to the study of fear are experiencing a resurgence (reviewed in ref. 22). For instance, Choi and Kim^23^ introduced a task that reproduces natural foraging conditions. In this task, hungry rats are confronted with a mechanical predator, called Robogator, when they leave the safety of their nest to forage in an elongated arena (Fig. 1a). The predator is threatening: when rats come near, it surges forward and repeatedly snaps its jaws shut. In control conditions, rats show many signs of apprehension in the foraging task (Fig. 1b, c). Before initiating foraging, they wait for ~20 s at the edge of the foraging arena. Choi and Kim observed that following amygdala inactivation, rats ignore the Robogator, as if they have become fearless. On the surface, this result agrees with the view emerging from fear conditioning studies: since the amygdala contains neurons that signal threat and in turn generate defensive behaviors, inhibiting the amygdala should abolish defensive behaviors in the foraging task. However, subsequent unit recordings from the basolateral (BL) nucleus of the amygdala are not consistent with this explanation.

Recording BL neurons, Amir *et al.* ^24^ distinguished two types of projection cells in the foraging task, with the incidence of the first (Type-1) being 10-fold higher than that of the second (Type-2) and the two exhibiting inverse activity fluctuations in most conditions (Fig. 1d). During the waiting period prior to foraging, the activity of the prevalent Type-1 cells matched predictions of the threat-coding model. That is, their firing rates (FRs) were higher when rats seemed more apprehensive: i.e., when they decided to abort rather than initiate foraging (Fig. 1e, *blue*) or in the presence of the predator (Fig. 1f, *blue*). Yet, during foraging, the activity of Type-1 cells was opposite to that predicted by the threat-coding model: their FRs were markedly *reduced*, becoming nearly silent near the predator (Fig. 1d), even when they escaped. The activity of Type-2 cells was not related to threat either (Fig. 1e, f, *red*).

Some could argue that because rats were confronted with the Robogator multiple times, they eventually stopped fearing it. Arguing against this possibility however, the inhibition of Type-1 cells upon foraging initiation was also seen when the Robogator was first introduced (Amir and Paré, unpublished observations). Others might offer a different interpretation for these findings. For instance, they might point out that when rats did not venture out of their nest, they were afraid and thus BL neurons fired. And if rats were so bold as to initiate foraging despite the proximity of the predator, they were presumably not afraid at that instant and BL activity decreased. While this account seems to fit the threat-coding model, it cannot explain why BL cells were nearly silent when rats actively escaped the predator. Surely, the predator must be threatening since rats run away from it.

Furthermore, the threat-coding model cannot account for the most surprising finding to emerge from the Amir *et al*. study^24^: *task-related variations in BL activity were not directly driven by threats or rewards*. Indeed, Amir and colleagues compared the activity of basolateral complex of the amygdala (BLA) cells in the foraging task and two control tasks devoid of explicit threats or rewards (Fig. 1g). Unexpectedly, they found that across the three tasks, neither threat proximity nor reward availability predicted BL activity. Instead, Type-1 FRs varied with movement, generally showing increased activity during periods where behavioral output was suppressed and decreased activity during spontaneous exploration or food retrieval (Fig. 1g). Thus, the activity of BL neurons across a variety of tasks is not consistent with the dominant threat-coding model.

## A new conceptualization of the role of the amygdala

In a largely overlooked study, Jacobs and McGinty^25^ proposed that activity in the BLA promotes behavioral inhibition. Using unit recordings in freely moving cats, they took advantage of a spontaneous electroencephalographic rhythm at 12–16 Hz, the somatomotor rhythm (SMR), which predominates over the sensorimotor cortex and is associated with motor inhibition. They paired spontaneous SMRs with reinforcing electrical stimulation of the lateral hypothalamus to induce long periods of immobility during which BLA activity was increased. While purely correlational in nature, this observation led Jacobs and McGinty to suggest that BLA activity may promote behavioral inhibition.

This proposal is consistent with the results of Amir *et al.* ^24^ on BL activity. However, the term “behavioral inhibition” is problematic because it suggests that the amygdala promotes motor inhibition when the available data suggest otherwise. Indeed, on foraging trials where rats showed signs of hesitation, moving forward and backward and forward again, Amir *et al*.^24^ observed that Type-1 FRs correlated negatively with the speed of forward movements but increased during backward motion. Thus, it appears that BL activity does not promote motor inhibition per se but regulates something more general, which we refer to as behavioral engagement. We believe this designation is preferable because it accommodates the fact that in some cases disengaging from a situation also requires a motor output. The Amir *et al*.^24^ study therefore suggests the amygdala opposes behavioral engagement.

What do we mean by behavioral engagement? The term refers to a basic process that governs transactions between mammals and their environment: whether or not to engage with (or disengage from) stimuli or situations. It is a process akin to decision-making, although it is unclear whether it is deliberative in all species and conditions. This process is particularly critical for ethologically significant situations such as decisions related to foraging, interactions with conspecifics or confrontations with predators, in short problems the nervous system has evolved to address.

Clearly, a variety of factors (motivational, contextual, mnemonic, etc.) determine whether mammals engage with a situation and the amygdala is only one of many nodes taking part in processing, integrating, and weighting such factors.^26^ For instance, when deciding whether to forage or not, animals likely consider how hungry they are, the specific properties of the environment, the palatability of the food they might find there, whether they detect or previously encountered a predator in this environment, and so on. We propose that besides providing a continuous signal that regulates engagement, the amygdala allows the subject’s prior experience to enter into the equation. For instance, many studies have documented how inhibiting BLA activity reduces the impact of changes in US or reinforcer value.^27, 28^ Also, BLA activity enhances the consolidation of emotionally laden memories.^29^ Thus, activity-dependent plasticity in its own network or target regions might allow the amygdala to regulate a subject’s choice based on prior experience when deciding whether or not to engage with a situation or not.

The notion that BLA activity opposes behavioral engagement offers a new explanation for prior findings. For instance, the drastic increase in risk taking Choi and Kim^23^ observed in the foraging task after BLA inactivation may not be due to a reduction in threat signaling, but rather to reproducing the state of Type-1 firing suppression required to initiate foraging. Also, some of the unexplained consequences of amygdala lesions in monkeys, such as the urge to attend to all sensory stimuli and ingest non-food objects could be explained by a loss of engagement regulation by the amygdala rather than a reduction in fear. Similarly, the enhancement of social interactions seen in macaques with excitotoxic lesions of the amygdala^30^ might not be due to a loss of fear, but to a loss of engagement regulation. Supporting this interpretation, a recent study reported that muscimol infusions in the BL amygdala enhanced social interactions even in monkeys that were highly familiar with each other,^31^ ruling out the possibility that a loss of fear accounted for the enhanced social interactions.

## CS responses of amygdala neurons: from sensory inputs to behavioral outputs

In the 1990s, the model emerged that CS–US convergence in LA led to the Hebbian strengthening of CS synapses such that subsequent CS presentations excited LA cells more strongly, allowing them to elicit conditioned responses (CRs) via their projection to CeA.^6–9^ However, some aspects of this model were inconsistent with evidence available at the time. First, it was known that the most effective way to condition animals was to offset the start of the CS and US by 10–30 s with the two co-terminating, rather than presenting them simultaneously, as would be expected for a Hebbian mechanism.^32^ However, despite the persistence of the CS for tens of seconds, the CS responses of LA neurons were known to be transient: a brief burst of short-latency spikes followed by a return of FRs near baseline.^33–35^ Consistent with these observations, recent whole-cell recordings of BLA neurons in vivo revealed that prolonged auditory stimuli elicit synaptic potentials that rapidly become sub-threshold.^36^

Thus, by the time the US occurs, LA neurons are firing little, and synaptic inputs carrying CS information are no longer active. Yet, Hebbian plasticity should be contingent on N-methyl-D-aspartate (NMDA) receptor activity at the time of CS–US pairing. While the susceptibility of classically conditioned fear to NMDA receptor antagonists is consistent with this possibility,^37–39^ given the time course of LA activity and afferent inputs during the CS, it seems unlikely that NMDA receptors are engaged when the US is presented 20–30 s after CS onset.

Furthermore, fear conditioning was known to potentiate only the initial, short-lived response of LA neurons to the CS,^33–35^ raising the question of how such a transient CR could maintain CRs for tens of seconds. Despite these discrepant observations, the model lived on and it became natural to think of CS-triggered LA firing as sensory responses that automatically drive CRs. Soon, this tendency generalized to neurons in other amygdala nuclei and to appetitive conditioning paradigms.^40–45^

Yet, in the amygdala nuclei that are downstream of LA, like BL and CeA, CS-elicited firing parallels the time course of the associated CRs much more closely than in LA.^44, 46^ This raises the possibility that in between LA and its targets, there is a shift in the nature of the representation, from a *sensory code* to a *behavioral response code*. It seems to us that such a transformation is needed to account for the behavioral flexibility displayed by mammals when threatened. For instance, depending on threat proximity, rats will display freezing, fleeing, or attack.^47^ Models of fear learning solely based on the potentiated sensory responses of LA neurons cannot account for such flexibility.

**Fig. 2:**
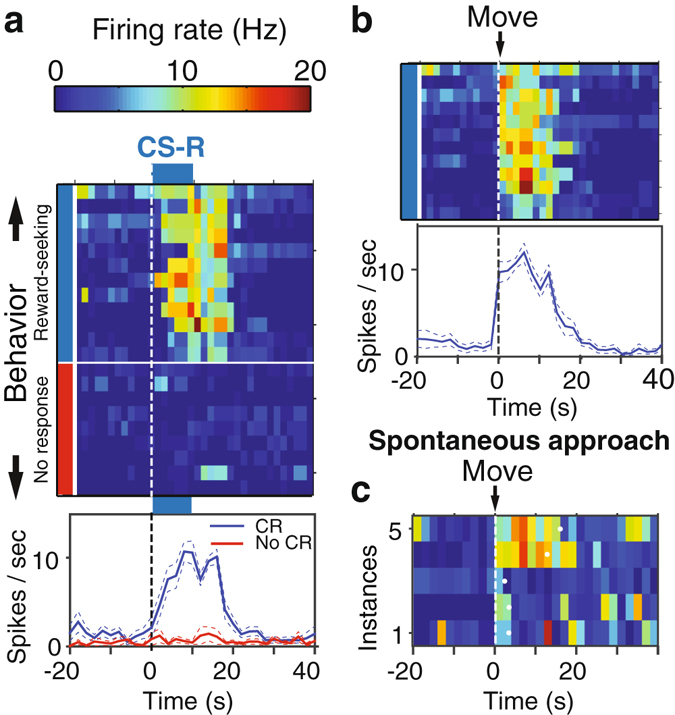
Activity of BL neurons during CSs varies depending on behavior. Rats were presented with two CS, one of which predicted reward delivery (CS-R) and the second (CS-N), a neutral outcome. After rats learned to retrieve the liquid reward at the conclusion of the CS-R, the two CSs were presented in random order while recording BL neurons. **a** Activity of a principal BL cell during multiple presentations of the CS-R. Activity is depicted as a color-coded raster (*top*) and peri-CS histogram of neuronal discharges (*bottom*; *solid* and *dashed lines* are averages and SEM, respectively). Trials are grouped by behavior as indicated by the *colored bar* on the *left* of the rasters (*blue*, approach of water-port; *red*, no approach). Rasters and peristimulus histograms in **b** depict the activity of the same cell as in **a**, but aligned to onset of water-port approach instead of CS onset. Note higher variations in firing latency when activity is referenced to onset of CS-R than to approach behavior. **c** Raster and histogram show activity of the same cell when the rat spontaneously initiated approach of the water-port in the absence of CS. *White dots* in raster indicate time when rats left the water-port. *Source*: Ref. 48

If indeed the CS representation shifts from a sensory code in LA to a behavioral response code in downstream nuclei, given a CS–US contingency determined by the investigator, firing during the CS should be variable in targets of LA, depending on trial-to-trial variations in the subject’s “decisions”, even though the CS–US contingency is fixed. Lee *et al*.^48^ examined this question by contrasting the activity of BL neurons when rats did, or did not, produce the appropriate CR (Fig. 2). One tone (CS-R) predicted reward delivery while another (CS-N) did not. As a result of conditioning, a low proportion of projection cells (hereafter termed R-neurons) exhibited increased FRs during the CS-R. Lee *et al*.^48^ found that the CS-related firing of R-neurons varied strongly with conditioned responding: it was present when they emitted the conditioned approach behavior in response to the CS-R (Fig. 2a, *blue*) but was absent when rats omitted it (Fig. 2a, *red*). Furthermore, R-neurons responded to the CS-N when rats exhibited (in error) the CR during the CS-N. Therefore, the activity of R-neurons is only coincidentally related to the CSs’ sensory properties. They actually encode behavioral output (Fig. 2b, c). Presumably, amygdala nuclei downstream of LA contain multiple subsets of neurons that drive different aversive or appetitive behaviors via segregated projections to various subcortical sites.^49–53^

On the surface, the existence of BL cells that are preferentially active during specific behaviors (like R-neurons) appears inconsistent with the notion that amygdala activity inhibits behavioral engagement. A possible solution to this conundrum is that the ability of BL to influence behavior not only depends on the activation of a specific subset of behavior-coding neurons but also on the suppression of all others. Consistent with this possibility, in a mixed appetitive-aversive conditioning paradigm, only 7% of principal cells increased their FRs when the appetitive CS was presented; most (52%) were inhibited (S.C. Lee, A. Amir, D. Haufler, D. Paré, unpublished observations). Although excitatory CS responses tend to attract more attention, inhibitory responses actually prevail, consistent with our model.

So far, there were few opportunities to explore these questions because investigators have relied heavily on classical fear conditioning, where the range of possible behaviors is extremely limited. Clarifying what amygdala neurons code for will require behavioral paradigms that allow one to compare the activity of the same neurons when animals express a larger repertoire of spontaneous and learned behaviors under multiple reinforcement contingencies. Parallel measurements of autonomic responses, such as pupil dilation, gastric motility, as well as heart and respiration rate, would also be useful.

## Gatekeepers of the amygdala

**Fig. 3:**
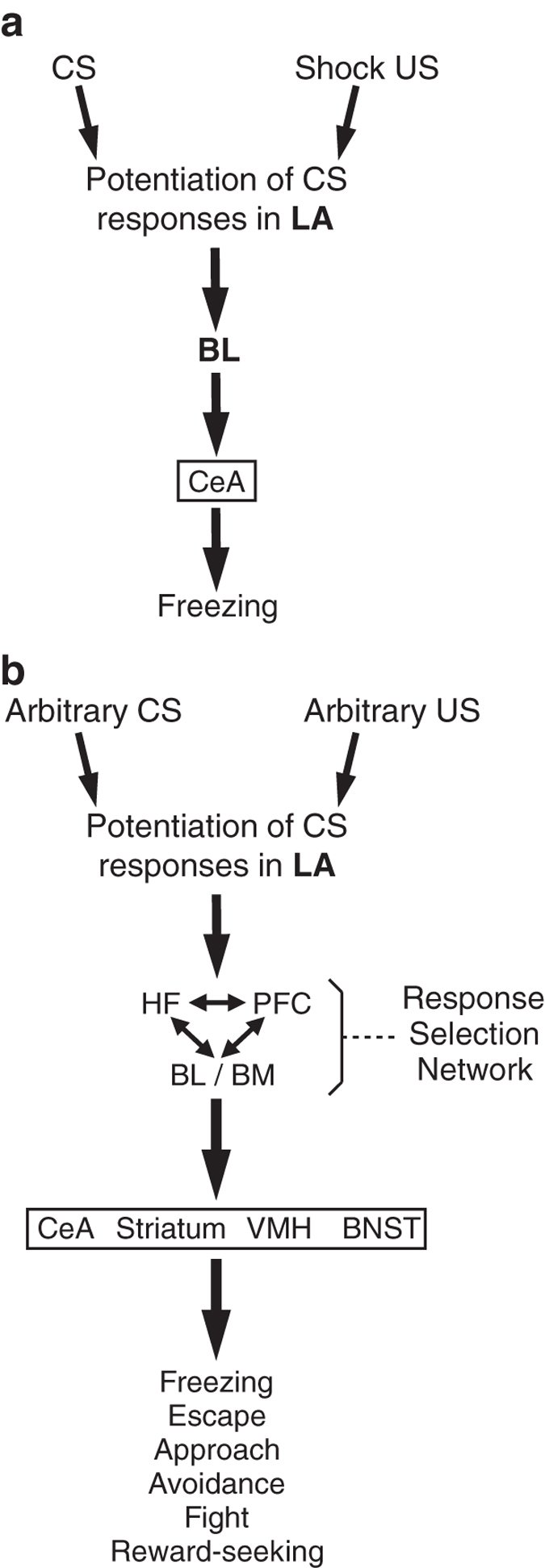
Schema of connections through the amygdala. **a** View based on fear conditioning. Convergence of tone CS and shock US potentiates tone responses of neurons in the LA, which then activate BL amygdala neurons to drive freezing via the CeA. **b** Beyond fear conditioning. Arbitrary CS–US pairings potentiate CS inputs to LA neurons, which project to a network of interconnected structures, including BL, basomedial nucleus (BM), mPFC, and HF. This network selects an appropriate response from a variety of possible alternatives. Responses are executed through descending projections to CeA, striatum, ventromedial hypothalamus (VMH), or bed nucleus of the stria terminalis (BNST)

The above indicates that in between LA and downstream amygdala nuclei is a fundamental shift from a primarily sensory representation to a behavioral response code. How does this transformation occur? The strong relation between BL activity and behavior irrespective of CS identity^48^ suggests that the impact of CS-related inputs from LA to BL can be overridden by other inputs to BL neurons. Prime candidates for this gating role are the medial prefrontal cortex (mPFC) and associated cortical regions (Fig. 3). Indeed, two mPFC areas, the prelimbic (PL) and infralimbic (IL) regions, are reciprocally connected with BL.^54–56^ Moreover, like BL, they form strong bidirectional connections with high-order cortical regions like the rhinal cortices and hippocampal formation (HF), directly^54, 57–59^ or via midline thalamic nuclei.^60^ Intriguingly however, although they exert an inverse influence on CRs,^15, 61^ depending on the task, say drug-seeking vs. fear conditioning paradigms, PL and IL, respectively, facilitate or inhibit appetitive and aversive responses.^62^

There are a number of similarities between PL and BL. Similar to BL neurons, the CRs of PL neurons are sustained throughout a 30 s CS and correlate with levels of freezing.^63^ Similar to BL, the expression of conditioned freezing is reduced by pharmacological^64–66^ and optogenetic^67^ inactivation of PL. Moreover, fear conditioning increases AMPA receptor function at PL synapses onto BLA neurons, thereby augmenting the responses of BLA neurons to the CS.^68^

**Fig. 4:**
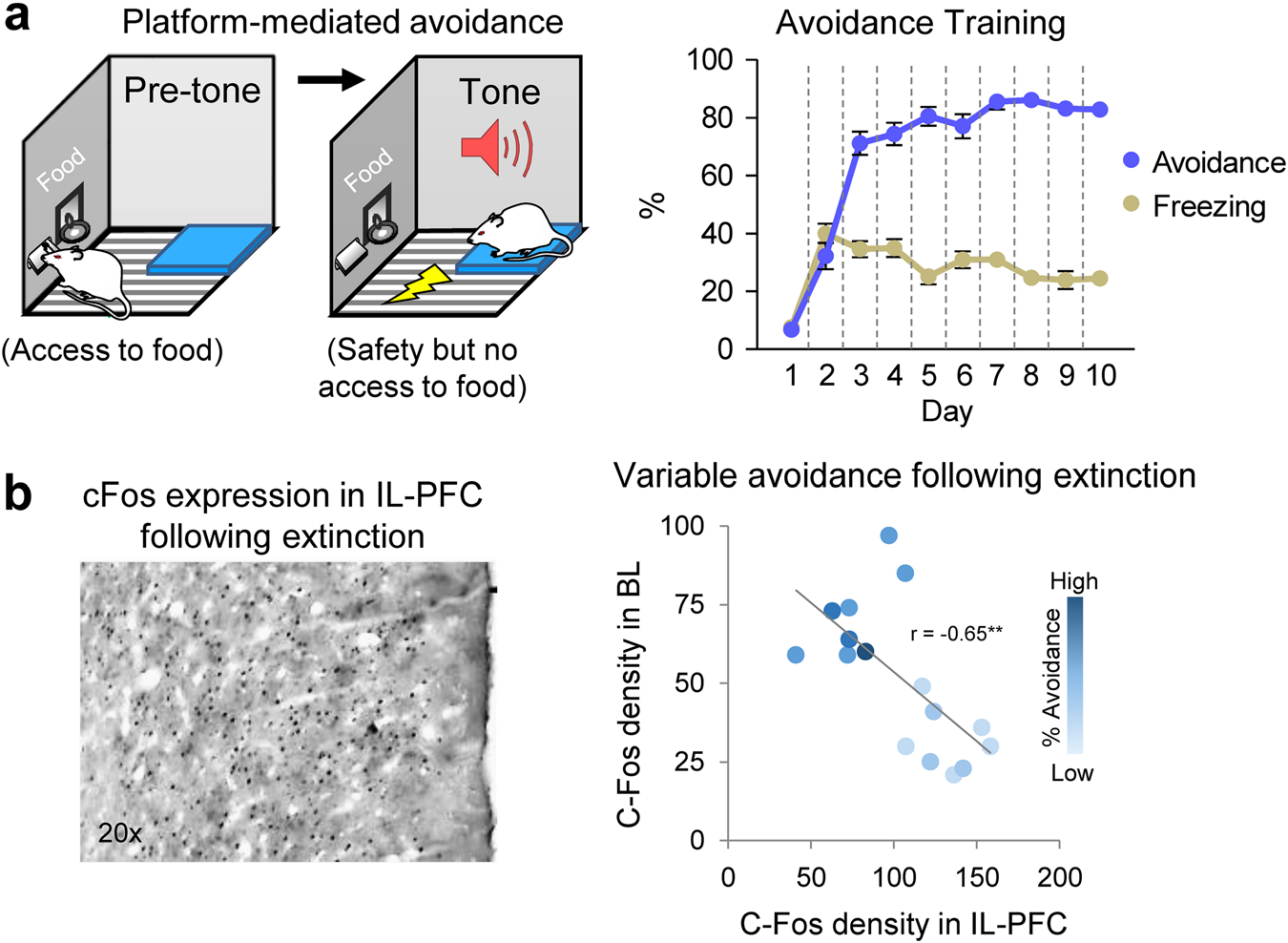
Platform-mediated avoidance task. **a** Food-deprived rats pressing a bar for food are exposed to a tone paired with a footshock. Over several days of training, rats learn to avoid the shock by stepping onto a platform on the opposite side of the chamber far from the food bar (conflict). As avoidance is acquired, freezing to the tone (fear) is reduced. *Source*: Ref. 72. **b** Following 2 days of extinction training (tones without shocks), most rats cease avoiding and remain at the food bar when the tone is presented. However, some rats continue to avoid. The immediate early gene cFos, an activity marker, reveals that rats continuing to avoid have elevated activity in BL and reduced activity in IL-PFC. Similar to the Robogator task above, BL activity is associated with lack of movement toward food, perhaps due to a failure of inhibition by inputs from IL. *Source*: Ref. 73

While these findings have been interpreted as PL triggering fear-induced freezing by exciting BL neurons,^15^ it is also possible that PL activation of BL triggers behavioral disengagement. Consistent with this, lesions or inactivation of BLA block active avoidance responses in the shuttle avoidance paradigm, where rats must move to an adjacent chamber to avoid being shocked.^69–71^ Such avoidance responses resemble the backward movements away from the Robogator which are correlated with increased BL activity (see above), and can be seen as an example of behavioral disengagement. In a platform-mediated avoidance task that pits shock avoidance against retrieval of food, pharmacological inactivation of PL reduces expression of avoidance but has *no effect* on freezing.^72^ Furthermore, inactivation of IL impairs extinction of this type of avoidance. Similar to the Robogator task, but differing from shuttle avoidance, platform-mediated avoidance involves opposite response tendencies (avoiding vs. feeding). Consistent with the behavioral engagement model, rats failing to extinguish platform-mediated avoidance (remaining on the platform despite the absence of shock) show elevated activity in BL as well as reduced activity in IL^73^ (see Fig. 4), reflecting persistent disengagement from food-seeking. PL neurons show excitatory responses to tone onset and platform mounting in this avoidance task (Diehl, Bravo-Rivera, and Quirk, unpublished observations), consistent with a role of PL in generating this type of avoidance. Surprisingly, however, naïve control rats show similar excitation of PL neurons by tones and platform mounting, suggesting that, similar to BL recordings discussed above,^24^ PL neurons may be signaling more general sensory and behavioral events rather than avoidance per se.

Herein lies the challenge of studying naturalistic tasks. Avoidance requires a decision by the animal to move away from threat, as does movement toward (or away from) food in the Robogator task. Movement-related correlates may or may not reflect decision processes related to food and threats. This differs markedly from the far simpler study of tone-evoked neuronal activity followed by reflexive freezing. It is far more complex (and interesting!) to characterize neuronal correlates of behaviors generated by the animal, than neural correlates of tones generated by the experimenter.

Perhaps BL contains multiple subsets of neurons, each regulating different behaviors. Presumably, in order for one of these opposite response tendencies to be expressed, most BL neurons must be inhibited and a specific subset activated. Paradigms that restrict the range of behaviors animals can express are inadequate to examine such a situation. So are pharmacological or optogenetic manipulations that non-selectively inhibit all neurons. Only through observation of neuronal activity during naturalistic behaviors will we be able to address these questions.

## Lessons learned

The above illustrates how a community of scientists, present authors included, can be biased by relying on an attractive, controlled laboratory paradigm. Amygdala researchers were motivated by their interest in the mechanisms of associative memory and emotional learning. They were understandably concerned with the need to achieve rigorous control over key experimental variables and to minimize the influence of confounding factors. This led to the adoption of a simple learning paradigm that restricted the range of possible conditioned behaviors. When a rat is presented with only one threatening stimulus in a testing box that allows for a single reflexive behavioral response, one is bound to find exactly what the experimental situation allows: neuronal responses that appear tightly linked to the CS and seem to obligatorily elicit the conditioned behavior. Only when the experimental situation allows for behavioral flexibility can one examine the nature of the relation between CSs, neuronal activity, decisions, and CRs.

Here, we are reminded of O’Keefe and Nadel’s wise recommendation that observational studies in naturalistic conditions should be performed prior to using controlled laboratory paradigms: “When we first venture into the unknown, we need not the incisive beam of the proud penetrating laser, but the gentle diffuse illumination of the humble torch. There will be plenty of time later for detailed investigation of the nooks and crannies: first we must find the mountains” (see ref. 74, p. 194).
